# Differences between stance and foot preference evident in Osprey (*Pandion haliaetus*) fish holding during movement

**DOI:** 10.1002/brb3.1126

**Published:** 2018-10-09

**Authors:** Laura L. Allen, Katie L Morrison, Wesley A. E. Scott, Steve Shinn, Alan M. Haltiner, Michael J. Doherty

**Affiliations:** ^1^ Department of Neurology Swedish Epilepsy Center Seattle Washington; ^2^ University Child Development School Seattle Washington; ^3^ Shinn Shots Los Angeles California

**Keywords:** Avian, footedness, foraging, laterality, raptor, symmetry, talon, tubercula

## Abstract

**Background:**

Skateboarders, snowboarders, and surfers all show stance preferences for which foot is forward while moving. We are unaware of other animals than humans with a stance preference, perhaps excepting Osprey, who fly their caught fish beneath them in a foot‐forward stance. We hypothesize there should be no difference between left foot forward, right foot back (conventional) versus right foot forward left foot back (goofy) stances or for fish holding with unilateral left or right foot. Online, publicly available, convenience images of Osprey catching fish were accessed and assessed by five independent reviewers using different Internet search engines or online photo series. Stance preference and footedness were tested using chi‐square analysis.

**Results:**

Stance preferences were evident with the left foot forward (conventional stance) on average 64–78% of the time (all *p *< 0.02). No difference in foot preference for either one‐foot grabs of fish during flight or for non‐flight nest/perch fish holding was evident.

**Conclusion:**

Flight stance of Osprey holding fish shows a lateralized preference in a proportion similar to skateboarders of surfers. We discuss stance preferences in the setting of complex movements and potential flight and survival advantages for Osprey.

## 1 BACKGROUND

Lateralized and localized brain functions permit complex actions to occur at the same time. In humans for instance, speech reception and hand motor areas have little anatomic overlap with areas responsible for vision or touch sensation, yet all can function simultaneously. Lateralized brain functions permit multi‐tasking, and help facilitate communication, precise movements, and intelligence (Rogers, Vallortigara, & Andrew, 2013). Left and right differences in brain function are not unique to humans. In birds, flight is a particularly complex movement, presumably aided by lateralized brain functions.

Lateralization in birds is well studied, at least in studies of footedness during feeding and perching behaviors. From studies of raptors, Goshawks and Marsh Harriers may show preferences for food‐holding with the left leg (Bond, 1942; Hosking, 1943). Parrots prefer the left foot for food holding and the right for perching (Harris, 1989). Perhaps more interesting yet, again from the study of parrots, the birds with lateralized foot preferences displayed better bird vocabulary, suggesting intelligence and lateralized abilities are linked (Snyder & Harris, 1997).

Avian footedness may arise to assist postural and positional controls (Rogers et al., 2013). Yet patterns of lateralized avian preferences during flight and complex movement like foraging are not as well studied. Budgerigars when presented with a left or right oriented hole to fly through under threat consistently had individualized preferences for the left or right flight path‐ but not both‐ in finding their escape routes (Bhagavatula, Claudianos, Ibbotson, & Srinivasan, 2014). A male short‐eared owl preferred to fly with voles dangling from the left foot in 12/13 sorties (Dudley, 2011). Osprey (Pandion haliaetus) based on return‐to‐nest behavior held prey more often in the right foot (Marie, 2004). The latter is the only observation we found of Osprey foot preferences, though we believe Osprey may be ideal for studying lateralized preferences during flight.

**Figure 1:**
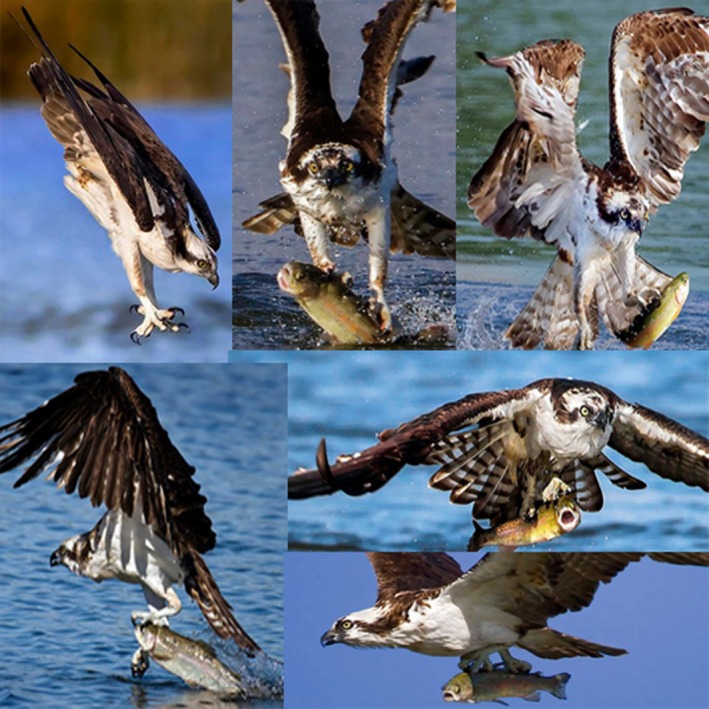
Osprey (Pandion haliaetus) hunting and capturing trout, clockwise from top left: showing steep‐angled attack dive, talons forward of head; A two‐foot grab is noted on bird‐alighting from water with right foot forward and front talon ahead of first dorsal fin while fish is in the midst of subcarangiform escape motions; a left foot grab with fish in motion; a sequence of two stances in‐flight, with left foot forward preference; and a left foot forward one‐foot grab at water's surface with talon position ideal for pithing through the trout's eye and cranial vault. (Photo credit: Steve Shinn)

Osprey hunt *live* fish, their aerial attack occurs in spectacular steep‐angled dives, talons forward, wings tucked (Figure 1). Dives may end with the Osprey completely submerged. Dive success in one review varies between 44 and 48%, with average fish size of 28 cm (Cutthroat trout at Yellowstone Lake, Wyoming, USA) (Swenson, 1978). Standard weigh equations for Yellowstone cutthroat, suggest a 28 cm fish would weight 227 grams, alternatively in Washington state, an average 28 cm coastal cutthroat‐ based on a 729 fish series, weighed 234 grams (Kruse & Hubert, 1997; J. Losee, Washington Department of Fish and Wildlife, personal communication, 2018).

Live fish are grabbed with talons rather than the beak. Examples of Osprey predation sorties and foot holding are perhaps most accessible through online film review (Wildscreen Archive, 2012). Osprey emerge from the water by flapping their wings, holding the still‐alive fish beneath them. Fish repositioning may occur in the water or shortly after taking flight, as might an in‐flight rotatory head and body shake to dry the bird. During flight, the preferred fish position is with head facing forward, aligned under the Osprey, head to tail. To stabilize the fish, one talon often grabs close to or even in midline cranial structures of the fish, while a second talon grips close to the dorsal fin. Positioning of one foot in front of the other and along the midline of both fish and bird means an in‐flight stance preference occurs (Figure 1), *one foot must be in front of the other*. On arrival to nest or perch, Osprey typically land with one talon no longer in the fish, they then hold the fish down with the remaining talon to eat. Osprey feed by preferentially devouring the fish head first, in so doing decapitating the fish. Osprey often alight and fly with the headless fish from perch back to nest, where feeding resumes.

Because Osprey assume a *stance* with fish holding during flight, study of lateralized preferences for which foot is forward, which is back can occur. The purpose of this study is to assess foot preferences of Osprey in flight or otherwise.

## 2 METHODS

Using common Internet search engines (Google, Bing, Yahoo, DuckDuckGo) and the search term “Osprey catching fish” a minimum of 50 pictures of different Osprey were assessed by four observers, one observer per search engine, for stance preferences while holding fish during flight. Foot preferences were also noted if the fish was held by only one talon and fish orientations with head forward or not were assessed. Images were not used if foot orientation was unclear, if the same location *or* photographer was credited for the photo, or if more than one fish was caught. Exceptions were made if the Osprey was clearly identifiable with unique leg banding or clearly discernable markings suggested a different bird, in which case additional birds could be studied from the same location or photographer.

In order to assess unique data that *did not* potentially overlap with the other Internet searches, a fifth data set was studied, using new and unique images. Instead of keyword searches, sequential images of Osprey were studied from a photo‐sharing group site called *Ospreys Only* Ospreys Only Group, 2018). This site allowed contact with the photographers, 119 of whom were queried as to whether or not their image was flipped or inverted to verify the stance or foot preference they uploaded was accurate. In addition, images were classified for secondary outcomes as to whether or not they were close to 30 cm or less off the surface of the water; if the fish was headless, how was it held; or what foot the osprey preferred to hold fish in while on nest or perch. Images were assessed by sequential study working backwards from May of 2018 to August of 2017. Statistical testing was performed using two‐tailed chi‐square with foot preferences assumed to be equally distributed.

## 3 RESULTS

Results are seen in Table 1. Across all observers, fish‐holding *stance* preferences were noted in all series for left foot forward, right foot back (64–78% of images surveyed, all *p* 0.02 or less). Conventional fish positions were fish head facing forward (Ospreys Only dataset [OOD] *n* = 112 facing forward, *n* = 10 facing backwards) and aligned beneath the body of the bird. Of the images showing the fish held with the head facing backwards, five had the fish less than a foot off the water and one was landing at a nest, suggesting that mid‐flight preference is by far for the fish to be facing forward.

**Table 1:** Survey results including images accessed through varied Internet search engines and from review of Ospreys Only Dataset

	Google	Bing	Yahoo	DuckDuckGo	Ospreys only
Two‐foot grabs					
Left/right	44/25	28/11	36/10	23/10	49/27
Percent	64	72	78	70	64
Chi‐square	5.2	6.4	14.7	5.1	6.36
*p*‐value	0.02	0.01	<0.01	0.02	0.01
One‐foot grabs					
Left/right	9/13	6/7	10/9	10/11	23/23
Percent	41	46	52	47	50
Chi‐square	0.7	0.08	0.05	0.05	0.00
*p*‐value	0.39	0.78	0.82	0.83	1.00

Leg preference was *not* evident with one‐foot holds, though the gripping talon was preferentially in front of or spanning the dorsal fin with one‐foot grabs on average 72% of the images (Table 1). One‐foot preference in fish holding was most commonly located in anterior fish segments, at or just in front of first dorsal fin. The majority of fish captured appeared to be bream, shad, trout, goldfish and less commonly needle or flatfish.

In the OOD, 119 photographers were surveyed as to whether or not their images were flipped or inverted or rotated, 82 responded (69% responder rate) of those, *none* altered their images in a manner that would invalidate foot or stance preferences. Two of the photographers from Ospreys only had different photos also assessed in the Internet search‐based assessments, otherwise photographers and photos in this set were unique.

From the OOD, One‐foot grabs were evident in 46 of the pictures with the breakdown of left to right at 23 each (Table 1). Ninety‐eight of Osprey in flight images were greater than 30 cm. above water. Twenty were around 30 cm. or less from the water's surface and of those, seven were unilateral grabs (left 3, right 4) and on the remaining 13 photos of two‐foot grabs, stance was left‐forward: right‐forward at 8:5.

Interestingly, 22 pictures show Osprey carrying *headless fish*, which Osprey prefer to carry with one foot, with a left/right breakdown of 9/12 (chi‐square 0.43, *p* = 0.52), only one picture showed an Osprey using two feet for flight with a headless fish.

Not‐in‐flight foot positions from OOD while perched or in nest holding fish showed a breakdown of 19 left and 19 right holding fish. Only one photo of an osprey on a beach (an atypical resting location compared to the other perch or nest shots) showed bilateral foot holding.

## 4 DISCUSSION

Osprey showed no foot preferences for in‐flight one‐foot grabs, or for not‐in‐flight fish holding at the perch or nest. We were unable to replicate prior raptor work showing footedness of harriers, goshawk owl, or osprey (Bond, 1942; Dudley, 2011; Hosking, 1943; Marie, 2004). However, during complex movements involved in flight‐with‐prey, stance preferences for the left foot forward were consistent and reproducible (Table 1). We are unaware of animals or birds *other than humans* that display stance preferences during travel or movement. These findings suggest a lateralized brain function likely underpins Osprey flight stance, but apparently *not* footedness.

In board‐in‐motion sports, humans choose a “regular” stance with the left foot forward or “goofy‐foot” stance with the right foot forward (Furley, Dörr, & Loffing, 2018). Breakdowns include surfers 65% left‐forward/35 right‐forward, skateboarders 56% left‐forward/44 right‐forward and skilled snowboarders 66% right‐forward/34% left‐forward (Furley et al., 2018; Staniszewski, Przemyslaw, & Wiszomirska, 2016; Warshaw, 2005; Willa, 2013). The Osprey have a 68/32 split of left‐forward/right‐forward stance that is very similar to skateboarders or surfers. Elite snowboarders, and not osprey, appear to be the outliers in stance preference (Staniszewski et al., 2016).

In humans, board stance preferences become more complex with the skill of the rider. For instance, surfers show stance preferences but also have preferences as to how they face the breaking wave, typically preferring to face the wave in a frontside position, rather than away from it in a backside position (Furley et al., 2018). Additionally, elite surfers competitive success depends on how well they ride waves that jeopardize preferred frontside and conventional stances (Furley et al., 2018). Skateboarders riding bowls show forward *and* backward motion‐ without stance change‐that is in the same ride a conventional *and* goofy stance may be assumed depending on travel direction as opposed to a conventional, more consistent racing/riding position. In both surfers and skaters, the implication of complex sensory inputs, particularly vision and direction of travel, directly influences stance. Following on that idea, in an elegant discussion of stance from studying snowboarders, Staniszewski argues that balance required to execute turns and maintains stance control requires entire motor system involvement, and as such lateralized preferences for footedness to explain stance during critical balance exercise where arms, axial positioning, or even head tone adjustments are also used is simplistic (Staniszewski et al., 2016).

We hypothesize the laterality of stance preference relates directly to the complexity of Osprey movements. Osprey may carry their partially consumed dead headless fish with one foot from perch to nest, without preference for which foot is used. As opposed to a live fish, the dead fish is a relatively predictable load, particularly when flying from perch to nest. Yet to secure a live, ~230 gram fish, emerge from water, take off and successfully fly must necessitate an extraordinary and rapid integration of load, balance and forward motions. We suspect the Osprey's lateralized stance preferences occur due to two main reasons, controlling fish motion and potential kleptoparasatism during flight.

For two‐foot holds, front talon locations over/in the fish's cranial vault potentially expedite pithing (Figure 1). In other raptors, talon grips can change, presumably to help kill pray (Bond, 1942). Like Owls, an Osprey's outer talon is reversible in orientation, which presumably permits grasping, orienting, and killing of fish across a broader range of rotations/positions (Terres, 1980). Talons serve as the sharp end of gripping, as the very end stage of orthodromic motor functions. However, if Osprey talons are analogous to human fingers, perhaps they also permit joint position sense, and in so doing initiate relays of prey weight *and* motion back to the brain. Independent of talons, Osprey feet are additionally lined with special tubercula, postulated mainly for fish‐gripping (Terres, 1980). Presumably, talon *and* tubercula maximize grip with fish‐forward orientations, but might they *both* also act as sensory inputs about fish motion and load?

Fish move in wave‐like movements. The fish studied for this survey, the majority of which seemed to be trout, shad, goldfish, or bream (alternatively none were fast‐swimming deeply‐forked tail pelagic fishes), have a subcarangiform pattern of movement that is the rear‐half of the fish is responsible for most‐ but not all‐ propulsion (Figure 1). Two‐foot placements, perhaps through talon and tubercula inputs, probably help the Osprey define fish center of gravity. Furthermore, front foot preference for fish holding appears to be close to the head, while the rear foot is closer to the first dorsal fin. If the head position helps pith and kill the fish, the first dorsal position is likely in an area that is less likely to move. If the fish moves in a hinge‐like movement, the rear most foot might prefer to cover the pin or least variable center of the hinge‐movement. This would confer advantages in predictable ways, such as removing the fish from under water, emerging from currents, flying into or against winds, minimizing fish writhing, or escaping kleptoparasitic behaviors from other birds.

The Snohomish river delta (Washington State, USA) hosts numerous summer Osprey. Bald Eagles frequent the delta, though in lower numbers than Osprey. The Eagles often attempt in‐flight hijacking of the Osprey's catch. During flight, Osprey are otherwise unable to defend from this kleptoparasitism particularly if talons are buried. Eagle pursuits can last for 5 min (Doherty, personal observation). The more agile the Osprey, the less agile the fish, the more likely the Osprey will escape an Eagle's pursuit. Kleptoparasitic behavior by Bald Eagles toward Osprey is otherwise well documented, photographic examples can be found online as well as in the early literature (Franklin, 1784; Shipper, 2015). In this 1784 correspondence Benjamin Franklin uses the term *fishing hawk* in describing Osprey:

For my own part, I wish the bald eagle had not been chosen as the representative of our country. He is a bird of bad moral character. He does not get his living honestly. You may have seen him perched on some dead tree, where, too lazy to fish for himself, he watches the labor of the fishing hawk; and when that diligent bird has at length taken a fish, and is bearing it to his nest for the support of his mate and young ones, the bald eagle pursues him, and takes it from him. With all this injustice, he is never in good case, but like those among men who live by sharping and robbing he is generally poor and often very lousy (Franklin, 1784).

In humans, there may be a frequency‐dependent selection survival advantage for handedness (Raymond, Pontier, Dufour, & Moller, 1996). Specifically, boxers may have performance advantages if they are left‐handed because their opponents are used to facing right‐handers. Whether similar advantages are conferred from Osprey stance preferences would perhaps depend on kleptoparasatic attack strategies, foraging behaviors relative to preferred feeding sites and their currents, prevailing winds, predominant directions of migratory and resident fish motion and schooling behaviors. Presumably, the only way to help tease out frequency‐dependent survival advantages would be with field study of foraging on smaller populations while accounting for those variables.

Concessions of our study include raters for the four Internet search engine datasets likely looked at similar bird pictures, meaning there is sample overlap between, but not within, raters. We do not know if stance is dependent on talon preference for fish pithing. We do not know if talons and tubercula have sensory functions, or if lateralized functions are still more complex. Examples of complex lateralizations during movement or flight might include if Osprey have a preference for wing or tail movements that favor one side. Perhaps the preference for one foot holding of headless fish is only statistically insignificant due to sample size. The OOD study is unique in that it does not overlap with prior images. There may be changes in Osprey foot positioning mid‐flight or due to adverse wind, current or flight conditions that would only be apparent with serial observation of the same bird(s) and appropriate wind and current direction data. We expect these concessions would randomize with assessments of multiple different birds in a diverse population.

## 5 CONCLUSION

Osprey show a stance preference for the left foot forward, right foot back only during flight They do not otherwise show foot preferences for flying with one foot holding fish, while flying dead fish or while not flying while holding fish. These stance preferences mirror that of skateboarders and surfers. This finding suggests complexity of movements during flight with potentially live prey are perhaps enabled through lateralized brain functions.

## CONFLICT OF INTERESTS

None declared.

## AUTHORS’ CONTRIBUTIONS

LA, AH, WS, KM, MD were responsible for assessing stance and foot preferences as well as drafting and revising the manuscript. SS was responsible for manuscript photos and revising manuscript. MD and AH were responsible for data analysis. All authors were able to view final manuscript.
